# Phytochemical Composition of Cranberry (*Vaccinium oxycoccos* L.) Fruits Growing in Protected Areas of Lithuania

**DOI:** 10.3390/plants12101974

**Published:** 2023-05-13

**Authors:** Rima Šedbarė, Sigita Sprainaitytė, Gintaras Baublys, Jonas Viskelis, Valdimaras Janulis

**Affiliations:** 1 Department of Pharmacognosy, Faculty of Pharmacy, Lithuanian University of Health Sciences, 50162 Kaunas, Lithuania; valdimaras.janulis@lsmuni.lt; 2 Kamanos State Strict Nature Reserve, 85354 Akmenė II, Lithuania; s.sprainaityte@yahoo.com; 3 Dzūkija Suvalkija Protected Areas Directorate, Group of Žuvintas Biosphere Reserve, 64351 Aleknonys, Lithuania; 4 Institute of Horticulture, Lithuanian Research Centre for Agriculture and Forestry, 54333 Babtai, Lithuania; jonas.viskelis@lammc.lt

**Keywords:** phenolic compounds, raw material, small cranberry, triterpenoids

## Abstract

The fruits of *Vaccinium oxycoccos* L. are an important source of bioactive compounds with antibacterial, antifungal, and anti-inflammatory effects. Studies on the phytochemical analysis of cranberry fruit samples showed that the qualitative and quantitative composition of biologically active compounds varied in cranberry fruit samples collected from different types of wetland sites: the total anthocyanin content was 698 ± 24–8352 ± 200 µg/g, the total flavonol content—518 ± 16–2811 ± 31 µg/g, the total content of triterpene compounds—4060 ± 122–6542 ± 157 µg/g, the content of chlorogenic acid—17 ± 0.4 µg/g to 1224 ± 41 µg/g, and the total content of proanthocyanidins—919 ± 19 µg EE/g to 3038 ± 137 µg EE/g. The percentage composition of anthocyanins in cranberry fruit varied between the different wetland sites: in some cranberry fruit samples, four anthocyanins (cyanidin-3-galactoside, cyanidin-3-arabinoside, peonidin-3-galactoside, and peonidin-3-arabinoside) were predominant, while in other samples, six anthocyanins (cyanidin-3-galactoside, cyanidin-3-arabinoside, peonidin-3-galactoside, peonidin-3-arabinoside, cyanidin-3-glucoside, and peonidin-3-glucoside) predominated. The results of these studies showed the differences in the composition of secondary metabolites in the studied cranberry samples and prove that the standardization of the qualitative and quantitative composition of cranberry fruit raw materials and the application of routine tests are necessary for the expansion of the use of botanical raw materials in the production of functional foods and phytopreparations.

## 1. Introduction

*Vaccinium oxycoccos* L. (syn. *Oxycoccus quadripetalus* Gilib., *Oxycoccus palustris* Pers.; *Ericaceae* Juss.), called small cranberry, bog cranberry, or European cranberry, is a perennial evergreen plant that is widespread in the natural coenopopulations of raised and intermediate wetlands in Europe, North America, and Asia [1,2,3]. The fruits of *Vaccinium oxycoccos* L. are harvested in European countries from their natural habitats and are used for food (juices, jams, jellies, sauces, and additives for meat products) and folk medicine to prevent urinary tract infections [2,4,5].

In the second half of the 20th century, land reclamation in Lithuania drained a large number of raised and intermediate wetlands, which reduced the natural habitat of cranberries [6]. The current peatland area in Lithuania is 352,000 ha, covering about 5% of the country’s territory [7]. Most of the wetlands are small, up to 50 ha in size, and are spread throughout Lithuania. The largest areas of cranberry bogs are found in the state-owned reserves of Žuvintas, Kamanai, and Čepkeliai [7,8]. Climate change and human activities are affecting sensitive wetland ecosystems, and the conservation and restoration of these areas is therefore important for the conservation of cranberry habitats [9,10].

Cranberry fruit samples were found to contain proanthocyanidins [11], phenolic acids [2,12], anthocyanins [1], vitamin C [13], triterpene compounds [14], and microelements and macroelements [15]. Studies on the bioactive compounds of small cranberry fruit, which determine their biological effects, are scarce and fragmented [2]. The literature contains descriptions of studies on the antibacterial [16,17,18], antifungal [19], and anti-inflammatory [20,21] effects of bioactive compounds in small cranberry fruit.

Since ancient times, people in Lithuania have followed the tradition of picking small cranberries in the high and intermediate wetland areas, using the berries in traditional dishes, drinks, and folk medicine. In order to substantiate the use of cranberries in scientific medicine and the development of pharmaceutical preparations, it is important to carry out detailed qualitative and quantitative studies on the composition of the biologically active compounds in cranberry fruit. The results obtained would enable the use of small cranberries (*Vaccinium oxycoccos*) as an alternative to the increasingly cultivated large cranberries (*Vaccinium macrocarpon*) due to their similar sensory properties and chemical composition [2,22]. It is relevant to carry out detailed studies on the qualitative and quantitative composition of biologically active compounds (anthocyanidins, phenolics, and triterpenoids) in cranberry fruit and to compare it with the chemical composition of the fruit of large cranberry cultivars grown in Lithuania. Phytochemical studies are relevant for the preparation of high-quality cranberry plant material and for the determination of the optimum time of picking of cranberries when the berries accumulate the highest amounts of biologically active compounds. The results are relevant for assessing the chemotypes of cranberry habitats and the status of cranberry bogs in Lithuania.

The aim of the study was to carry out a phytochemical analysis of cranberry fruits growing in the territories of the Lithuanian state-owned reserves Žuvintas and Kamanai, to determine the variation in the qualitative and quantitative composition of anthocyanins, flavonols, chlorogenic acid, proanthocyanidins, and triterpene compounds, and to evaluate the chemotypes of *Vaccinium oxycoccos* plants growing in natural habitats. The obtained data are relevant for the assessment of the most appropriate time for berry harvesting and for the clarification of the influence of the type of the trophic state of wetlands (oligotrophic, mesotrophic, or eutrophic) on the quantitative composition of biologically active compounds in cranberry fruits.

## 2. Results

Cranberry fruit samples were collected at different sites in the wetlands of Žuvintas and Kamanai reserves (Figure 6). Cranberry fruit samples were collected at the beginning of berry ripening (at the end of August 2021) and after the berries had reached ripeness (in October 2021). During the study, we carried out the qualitative and quantitative analysis of proanthocyanidins, anthocyanins, chlorogenic acid, flavonols, and triterpene compounds as well as the analysis of the phytochemical profile of the cranberry fruit samples collected from different sites.

**Figure 1:**
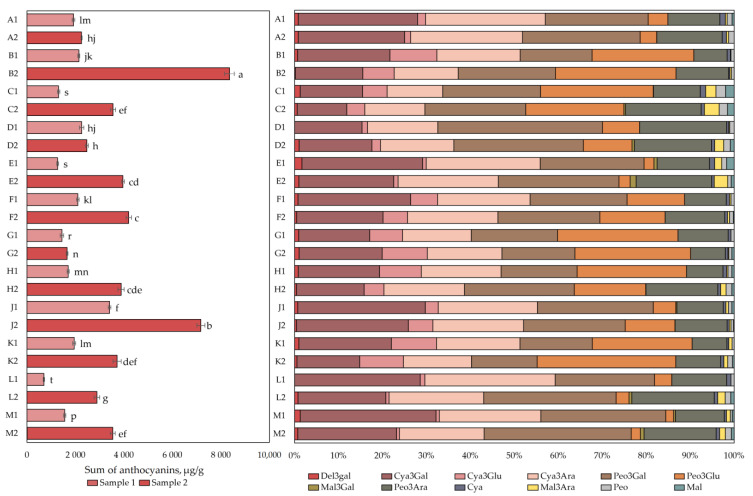
The content of anthocyanins and anthocyanidins in cranberry fruit samples collected from different habitats. A description of the cranberry fruit harvesting areas is given in Table 1. The number “1” next to the site letter indicates the first sampling time at the end of August (Sample 1); the number “2” next to the site letter indicates the second sampling time in October (Sample 2). Different letters indicate significant differences (*p* < 0.05) between the total amounts of anthocyanins in the tested cranberry samples. Del3gal—delphinidin-3-galactoside, Cya3Gal—cyanidin-3-galactoside, Cya3Glu—cyanidin-3-glucoside, Cya3Ara—cyanidin-3-arabinoside, Peo3Gal—peonidin-3-galactoside, Peo3Glu—peonidin-3-glucoside, Mal3Gal—malvidin-3-galactoside, Peo3Ara—peonidin-3-arabinoside, Mal3Ara—malvidin-3-arabinoside, Cya—cyanidin, Peo—peonidin, Mal—malvidin.

The qualitative and quantitative analysis of anthocyanins and anthocyanidins is presented in Figure 1. The total anthocyanin content in the cranberry fruit samples varied from 698 ± 24 µg/g to 8352 ± 200 µg/g. The highest total anthocyanin content (8352 ± 200 µg/g) was found in cranberry fruit samples collected in the oligotrophic-type wetland in Kamanai reserve (site B, Figure 1) in October (*p* < 0.05). The lowest total anthocyanin content (698 ± 24 µg/g) was found in cranberry fruit samples collected in oligotrophic-type wetland (site L) in Žuvintas reserve at the end of August (*p* < 0.05). Cranberry fruit samples collected in October showed up to four times higher total anthocyanin content (1649 ± 35–8352 ± 200 µg/g) than cranberry fruit samples collected at the end of August did (698 ± 24–3403 ± 51 µg/g). The increase in anthocyanin content in the cranberry fruit samples collected from the mesotrophic-type wetland in Kamanai reserve (site D) was not statistically significant (*p* > 0.05).

After analyzing cranberry fruit samples collected from different types of wetlands, they were divided into two groups according to the phytochemical profile of the predominant anthocyanins. The first group consisted of fruit samples collected in the mesotrophic (sites A and D) and oligotrophic (site E) wetlands of Kamanai reserve and in oligotrophic (sites L) and mesotrophic (site M) wetlands of Žuvintas reserve. Four anthocyanins, cyanidin-3galactoside, cyanidin-3-arabinoside, peonidin-3-galactoside, and peonidin-3-arabinoside, predominated in the fruit samples collected from these wetlands, accounting for 88.84% ± 4.2% of the total anthocyanin content (Figure 1). The second group included fruit samples collected in oligotrophic (sites B, F, G, and J), mesotrophic (site H), and eutrophic (site C) wetland types of Kamanai reserve and in the mesotrophic wetland type (site K) of Žuvintas reserve. Six anthocyanins were predominant in cranberry fruits collected from the abovementioned wetland types: cyanidin-3-galactoside, cyanidin-3-arabinoside, peonidin-3-galactoside, and peonidin-3-arabinoside accounted for 62.54% ± 7.52% of the total anthocyanin content, while cyanidin-3-glucoside and peonidin-3-glucoside accounted for 30.55% ± 7.17% of total anthocyanin content (Figure 1).

**Figure 2:**
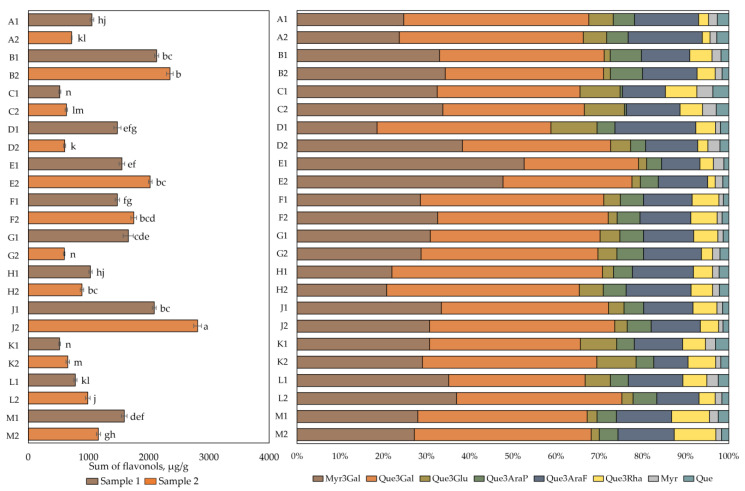
The content of flavonols in cranberry fruit samples collected from different habitats. A description of the cranberry fruit harvesting areas is given in Table 1. The number “1” next to the site letter indicates the first sampling time at the end of August (Sample 1); the number “2” next to the site letter indicates the second sampling time in October (Sample 2). Different letters indicate significant differences (*p* < 0.05) between the total amounts of flavonols in the tested cranberry samples. Myr3Gal—myricetin-3-galactoside, Que3Gal—quercetin-3-galactoside, Que3Glu—quercetin-3-glucoside, Que3AraP—quercetin-3-arabinopyranoside, Que3AraF—quercetin-3-arabinofuranoside, Que3Rha—quercetin-3-rhamnoside, Myr—myricetin, Que—quercetin.

The results of the qualitative and quantitative analysis of flavonol composition are presented in Figure 2. The total flavonol content in cranberry fruit samples varied from 518 ± 16 µg/g to 2811 ± 31 µg/g. The highest total flavonol content (2811 ± 31 µg/g) was found in cranberry fruit samples collected in the oligotrophic-type wetlands of Kamanai reserve (site J) in October (*p* < 0.05). The lowest total flavonol content (518 ± 16 µg/g) was detected in cranberry fruit samples collected in the mesotrophic-type wetland of Žuvintas reserve (site K) at the end of August. The quantitative composition of flavonols in fruit samples collected from this site was not statistically significantly different from the flavonol content in cranberry fruit samples collected from the eutrophic-type wetland site (site C) at the end of August or in cranberry fruit samples collected from the oligotrophic-type wetland site (site G) in October (*p* > 0.05).

Qualitative and quantitative analyses of cranberry fruit samples collected from the Kamanai and Žuvintas reserves confirmed that the phytochemical profile of flavonols was characteristic of cranberry fruit. The flavonol group compounds myricetin-3-galactoside (31.38% ± 7.68%) and quercetin-3-galactoside (38.33% ± 5.10%) dominated in the investigated cranberry fruit samples. Other compounds in the flavanol group were found in lower amounts. These were quercetin-3-arabinofuranoside (12.37% ± 2.41%), quercetin-3-rhamnoside (4.87% ± 2.03%), quercetin-3-glucoside (4.63% ± 2.84%), quercetin-3-arabinopyranoside (4.57% ± 1.60%), myricetin (1.82% ± 0.71%), and quercetin (2.02% ± 0.66%).

**Figure 3:**
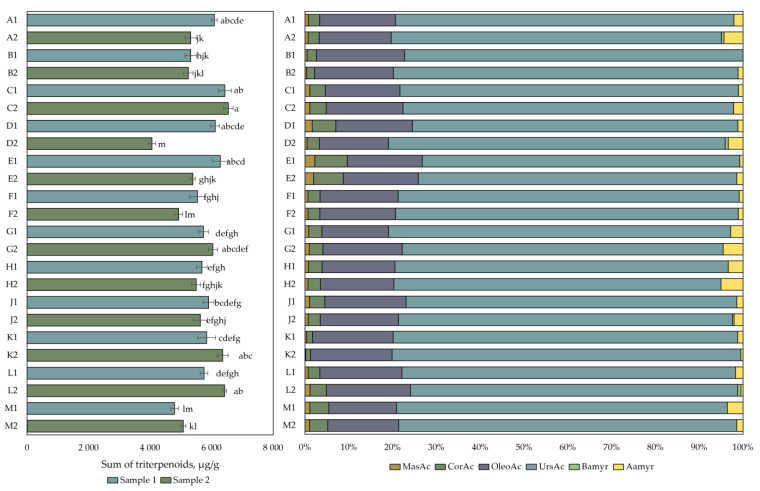
The content of triterpenic compounds in cranberry fruit samples collected from different habitats. A description of the cranberry fruit harvesting areas is given in Table 1. The number “1” next to the site letter indicates the first sampling time at the end of August (Sample 1); the number “2” next to the site letter indicates the second sampling time in October (Sample 2). Different letters indicate significant differences (*p* < 0.05) between the total amounts of triterpenic compounds in the tested cranberry samples. MasAc—maslinic acid, CorAc—corosolic acid, OleAc—oleanolic acid, UrsAc—ursolic acid, Bamyr—β-amyrin, Aamyr—α-amyrin.

The data of the qualitative and quantitative analysis of the composition of the triterpene compounds is presented in Figure 3. The amount of triterpene compounds in cranberry fruit samples varied from 4060 ± 122 µg/g to 6542 ± 157 µg/g. The highest total triterpene content (6542 ± 157 µg/g) was detected in cranberry fruit samples collected in the eutrophic-type wetlands of Kamanai reserve (site C) in October. The triterpene content of the fruit samples collected from this site was not statistically significantly different from the triterpene content of the cranberry fruit samples collected from the eutrophic (site C), mesotrophic (sites A and D), or oligotrophic (site E) wetland types of Kamanai reserve at the end of August or those collected from the oligotrophic (site L) or mesotrophic (site K) wetland types of Žuvintas reserve in October (*p* > 0.05). The lowest total amount of triterpene compounds (4060 ± 122 µg/g) was detected in cranberry fruit samples collected from a mesotrophic-type wetland vegetation (site D) in October (*p* < 0.05).

The analysis of triterpene compounds showed that ursolic acid was the most abundant in all the cranberry fruit samples, accounting for 76.24% ± 1.91% of the total triterpene content. The amount of oleanolic acid accounted for 17.44% ± 1.17% of the total amount of triterpene compounds in the studied cranberry fruit samples. The triterpene compounds found at lower total amounts were maslinic acid (0.91% ± 0.48%), corosolic acid (3.32% ± 1.49%), α-amyrin (1.98% ± 1.39%), and β-amyrin (0.10% ± 0.24%). Cranberry fruit samples collected from the oligotrophic-type wetlands of the Kamanai reserve (site E) showed higher levels of maslinic acid and corosolic acid, which accounted for, respectively, 2% and 7% of the total content of the triterpene compounds in the fruit samples of both collections.

**Figure 4:**
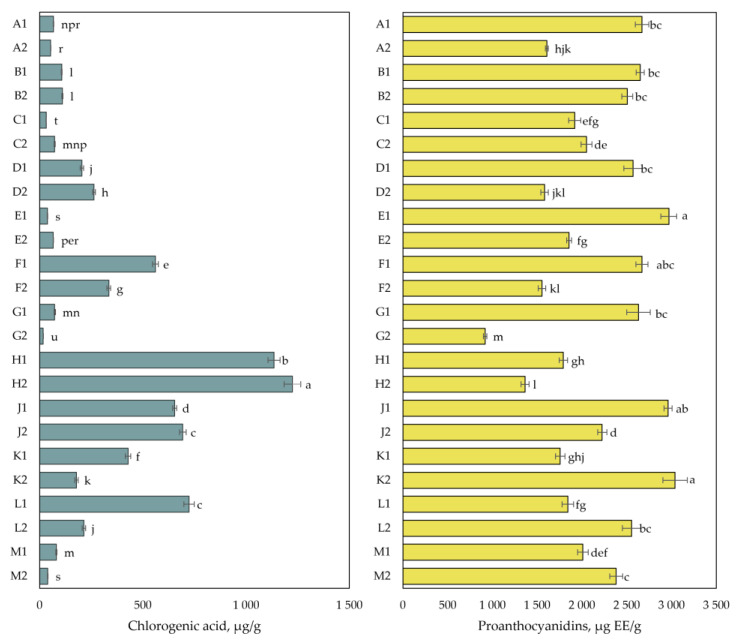
The content of chlorogenic acid and proanthocyanidins in cranberry fruit samples collected from different habitats. A description of the cranberry fruit harvesting areas is given in Table 1. The number “1” next to the site letter indicates the first sampling time at the end of August (Sample 1); the number “2” next to the site letter indicates the second sampling time in October (Sample 2). Different letters indicate significant differences (*p* < 0.05) between the amounts of the determined compounds in the cranberry samples tested.

The results of the quantitative analyses of chlorogenic acid and proanthocyanidins are presented in Figure 4. The amount of chlorogenic acid in cranberry fruit samples varied from 17 ± 0.4 µg/g to 1224 ± 41 µg/g. The highest total amount of chlorogenic acid (1224 ± 41 µg/g) was detected in cranberry fruit samples collected in the mesotrophic-type wetland of Kamanai reserve (site H) in October. The lowest chlorogenic acid content (17 ± 0.4 µg/g) was detected in cranberry fruit samples collected in the oligotrophic-type wetland of Kamanai reserve (site G) in October (*p* < 0.05).

The total amount of proanthocyanidins in cranberry fruit samples varied from 919 ± 19 µg EE/g to 3038 ± 137 µg EE/g. The highest total proanthocyanidin content (3038 ± 137 µg EE/g) was detected in cranberry fruit samples collected from the mesotrophic-type wetland (site K) of Žuvintas reserve in October, which was not statistically significantly different from the proanthocyanidin content found in cranberry fruit samples collected from the oligotrophic-type wetlands (sites E, J, and F) of Kamanai reserve at the end of August (*p* > 0.05).

**Figure 5:**
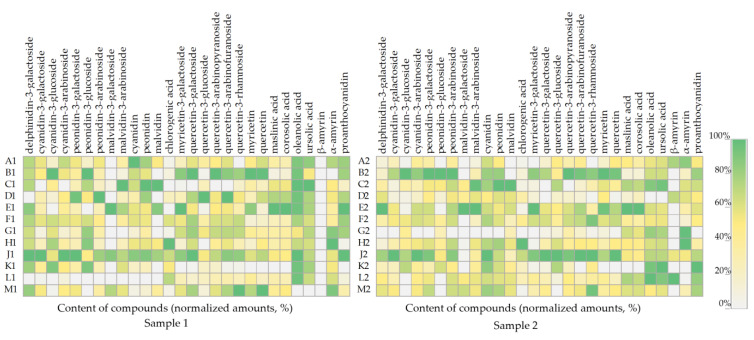
Heatmap of the amount (%) of individual constituents in cranberry fruits according to their growth site. The number “1” next to the site letter indicates the first sampling time at the end of August (Sample 1); the number “2” next to the site letter indicates the second sampling time in October (Sample 2).

The pattern of compound accumulation in cranberry fruit samples collected during the first (Sample 1) and the second harvesting (Sample 2) is shown in Figure 5. A comparative analysis of the obtained data showed that the cranberry fruit samples collected from the individual wetland sites showed a trend towards higher or lower levels of certain identified compounds. The model presented in Figure 5 shows that the percentage distribution of the compounds in the cranberry fruit samples of Sample 1, collected at the end of August, partially overlaps with the percentage distribution of these compounds in cranberry fruit samples of Sample 2, collected in October.

In the phytochemical composition model (Figure 5), the phytochemical composition of cranberry fruit samples collected in the oligotrophic-type wetland of Kamanai reserve (sites B and J) stands out. Samples from these sites (sites B and J) showed the highest levels of anthocyanins and flavonols during both the first and the second harvesting. Cranberry fruit samples collected from Kamanai reserve wetland sites (sites H and G) showed the highest levels of α-amyrin during both the first (respectively, 193 ± 15 µg/g and 162 ± 16 µg/g) and the second (respectively, 275 ± 22 µg/g and 273 ± 24 µg/g) harvesting. Cranberry fruit samples collected from the mesotrophic-type wetland in Kamanai reserve (site H) showed the highest levels of chlorogenic acid, as illustrated in the model shown in Figure 5. Cranberry fruit samples collected in the oligotrophic-type wetland of Kamanai reserve (site E) had higher levels of myricetin-3-galactoside, maslinic acid, corosolic acid, and malvidin and its derivatives malvidin-3-galactoside and malvidin-3-arabinoside, when compared to the levels of these compounds in cranberry fruit samples harvested from the other growing sites. Cranberry fruit samples collected in oligotrophic-type wetland (sites M, J, F, and B) had higher levels of quercetin-3-rhamnoside.

## 3. Discussion

The analysis of the qualitative and quantitative composition of biologically active individual compounds in *V. oxycoccos* fruit samples collected from different types of wetlands in Lithuania allows for comparing it with the qualitative and quantitative composition of biologically active compounds in fruit samples of *V. macrocarpon* cultivated in Lithuania. Anthocyanins found in *V. macrocarpon* fruit samples have been shown to increase the composition of good gut bacteria [23] and inhibit inflammatory processes in the gut [24], proanthocyanidins of type A have been shown to be effective in decreasing urinary tract infections [25], and triterpene compounds have anti-inflammatory effects [26] and inhibit the proliferation of cancer cells [27]. Comparative chemical composition studies are relevant for increasing the use of *V. oxycoccos* berries for health promotion purposes.

The phytochemical profile of the fruits of the small cranberry (*V. oxycoccos*) grown under Lithuanian climatic conditions was similar to that of the fruits of the previously studied large cranberry (*V. macrocarpon*) cultivars grown under Lithuanian climatic conditions [28]. The total anthocyanin content found in *V. oxycoccos* fruit samples during the ripening period was 698 ± 24–8352 ± 200 µg/g, while in the previously studied *V. macrocarpon* fruit samples, it was found to be 309 ± 15–9629 ± 266 µg/g [28]. The large variation in anthocyanin content during berry ripening is strongly influenced by genetic factors that regulate the mechanisms of anthocyanin synthesis in both cranberry species [29]. Karppinen et al. reported that during fruit ripening, the transcription levels of the anthocyanin biosynthesis-affecting enzymes chalcone synthase (CHS), dihydroflavonol 4-reductase (DFR), anthocyanidin synthase (ANS), and UDP-glucose flavonoid 3-*O*-glucosyltransferase (UFGT) are increased, leading to an increase in anthocyanin content of the fruit [30].

Light also affects the rate of anthocyanin synthesis in cranberry fruit [31]. Zhou et al. found that exposure to natural light increased the total anthocyanin content of cranberry fruit by 75.3% in 24 h and by 87.2% in 48 h compared to a control sample kept in the dark [32]. In our study, the increase in anthocyanin content was highest in the oligotrophic-type wetland habitats of Kamanai reserve (sites B, J, H, E, and F), possibly due to the more intense exposure to direct sunlight. These sites are open: plants such as *Carex lasiocarpa, Phragmites australis, or Eriophorum vaginatum* growing there do not block the sun, and that provides good conditions for the development and ripening of cranberry fruit.

The study found that the quantitative composition of anthocyanins varied between different natural habitats. Four anthocyanins (cyanidin-3-galactoside, cyanidin-3-arabinoside, peonidin-3-galactoside, and peonidin-3-arabinoside) were predominant in the cranberry fruit samples collected from Kamanai reserve wetland (sites A, D, and E) and from Žuvintas reserve wetland (sites L and M), while in the cranberry samples collected from sites B, C, F, G, H, and J of Kamanai reserve wetland and site K of Žuvintas reserve wetland, six anthocyanins (cyanidin-3-galactoside, cyanidin-3-arabinoside, peonidin-3-galactoside, peonidin-3-arabinoside, cyanidin-3-glucoside, and peonidin-3-glucoside) predominated. Vosra et al. investigated small cranberry fruit samples and found that ploidy 2× and 4× resulted in different percentages of anthocyanins arabinosides, galactosides, and glucosides in those samples [33]. They found that the tetraploid *V. oxycoccos* profile had larger amounts of cyanidin-3-galactoside (26.7% ± 6.1%), cyanidin-3-arabinoside (25.3% ± 2.7%), peonidin-3-galactoside, (24.5% ± 4.2%), and peonidin-3-arabinoside (17.3% ± 7.7%) and smaller amounts of cyanidin-3-glucoside (1.5% ± 1.1%) and peonidin-3-glucoside (4.7% ± 2.1%) [33]. The diploid *V. oxycoccos* chemotype was found to have larger amounts of cyanidin-3-glucoside (19.5% ± 6.5%), peonidin-3-glucoside (54.5% ± 9.6%), cyanidin-3-arabinoside (13.1% ± 4.8%), and peonidin-3-arabinoside (12.0% ± 8.3%) and smaller amounts of cyanidin-3-galactoside (0.5% ± 0.7%) and peonidin-3-galactoside (0.4% ± 0.8%) [33]. Česonienė et al. reported that the tetraploid type of *V. oxycoccos* showed a similar anthocyanin composition profile to that of *V. macrocarpon*, while the diploid type of the cranberry was characterized by a higher content of cyanidin and peonidin glucosides [34]. The genotype of small cranberries influences the quantitative composition of anthocyanins in fruits, and thus it can be concluded that different cranberry genotypes are present in the Žuvintas and Kamanai wetland habitats, which results in a variety of qualitative and quantitative anthocyanin compositions in cranberry fruits.

Another important group of bioactive compounds in cranberry fruits that determines the biological effects of fruit preparations are flavanols. A comparative analysis of the qualitative and quantitative composition of flavonols in *V. macrocarpon* and *V. oxycoccos* fruit samples showed that the total flavonol content (1465.56 ± 31.22 µg/g to 3688.52 ± 22.85 µg/g) in the large cranberry fruit samples varied more widely than the total flavonol content (518 ± 16 µg/g to 2811 ± 31 µg/g) in the small cranberry fruit samples [28]. In a study by Šedbarė et al., the quantitative composition of flavonols in *V. macrocarpon* fruit samples (quercetin-3-galactoside—29.07–33.87%, myricetin-3-galactoside—22.73–33.73%, quercetin-3-α-L-arabinofuranoside—14.55–19.88%, quercetin-3-rhamnoside—7.15–17.76%, quercetin-3-arabinopyranoside—2.13–3.44%, isoquercetin—0.98–4.73%, quercetin—0.91–3.80%, and myricetin—0.67–2.27%) was consistent with the quantitative composition of the flavonols found in fruit samples of *V. oxycoccos* [28]. Wang et al. investigated the flavonol composition of *V. macrocarpon* fruit samples and found that the amount of quercetin-3-galactoside was 31–46%, the amount of myricetin-3-galactoside—19–32%, the amount of quercetin-3-α-L-arabinofuranoside—7–17%, and the amount of quercetin-3-rhamnoside—7–14% [35]. These results are consistent with the quantitative flavonol composition found in the fruit samples of *V. oxycoccos* evaluated in our study.

The coefficient of variation for flavanols in the small cranberry fruit samples was 50.50%. In the cranberry fruit samples (sample 1 and sample 2), higher concentrations of flavonols were found in the samples collected from oligotrophic-type wetlands (sites B, E, F, and J), while lower concentrations were detected in the cranberry fruit samples harvested from eutrophic- and mesotrophic-type wetlands (sites A, C, D, H, and K). Cranberry fruit samples collected in October in the oligotrophic-type wetland habitat (site G) showed by 2.7 times lower flavonol levels than those found in cranberry fruit samples from the same site collected at the end of August. A 2.5-fold decrease in flavonols was also found in the cranberry fruit samples collected in the mesotrophic-type wetland habitat (site D). Changes in flavonol composition found in the other fruit samples collected from other habitats were not huge. Šedbarė et al. found that the total amount of flavonols in V. macrocarpon fruits halved during the ripening period (from 12 August to 22 October) [28].

The coefficient of variation of the total proanthocyanidin content in the tested cranberry fruit samples was 25.98%. Cranberry fruit samples collected in October in Kamanai reserve wetland habitats (sites A, B, D, E, F, G, H, and J) showed lower proanthocyanidin levels compared to the levels detected in the samples collected in late August. Cranberry fruit samples collected in October from Žuvintas reserve wetland habitats (sites M, L, and K) showed higher levels of proanthocyanidins compared to those detected in cranberry fruit samples collected in the same habitats at the end of August. Data from Wang et al. showed that similar (high/low) levels of proanthocyanidins in the cranberry fruit samples were related to genetic factors influencing flavonoid synthesis [35]. Yu et al. pointed out that the synthesis of proanthocyanidins is influenced by abiotic and biotic environmental factors (low temperature, UV radiation, exposure to fungal strains, etc.) [36]. Kamanai and Žuvintas reserves are about 250 km apart, thus these differences could be significantly influenced by both environmental conditions and the cranberry genotypes growing in the areas.

A comparative analysis of the total amount of proanthocyanidins showed that in *V. oxycoccos* fruit samples, it was 919 ± 19 µg EE/g to 3038 ± 137 µg EE/g and was lower than the total proanthocyanidin content of the *V. macrocarpon* fruit samples (2280 ± 220 mg EE/g to 8870 ± 570 mg EE/g) [28]. Narwojsz et al. investigated fruit samples of *V. macrocarpon* and *V. oxycoccos* and found that the total proanthocyanidin content of the samples differed little [22]. Jungfer et al. found that the content of A-trimeric proanthocyanins in *V. oxycoccos* fruit was by up to 15 times lower than that in *V. macrocarpon* fruit, and that differences in the qualitative and quantitative composition of proanthocyanidins might lead to differences in the efficacy of the species of *Vaccinium* in the treatment of urinary tract infections [37].

Phenolic (phenolcarboxylic) acids are an important group of biologically active compounds that determine the sensory and biological properties of fruit [38,39]. In the small cranberry fruit samples we studied, the greatest differences were found in the quantitative composition of chlorogenic acid (the coefficient of variation was 113.90%). The quantitative differences in the chlorogenic acid content presented in Figure 4 indicate that in some habitats, higher levels of chlorogenic acid were detected in cranberry fruit samples collected in August and in October. Šedbarė et al. found that chlorogenic acid content as a chemotype marker is specific to the studied *V. macrocarpon* cultivars Baifay, Howes, Pilgrim, Early Black, Red Star, and Stevens, and its levels remain constant during fruit ripening of [28]. The ability of *V. oxycoccos* fruits to accumulate chlorogenic acid, similarly to *V. macrocarpon* fruits, is determined by the genotype of the cranberry species, and can therefore also be regarded as a chemotype marker for *V. oxycoccos* fruits.

The content of triterpene compounds in cranberry fruit samples varied the least among the groups of the compounds studied, the coefficient of variation being 10.52%. The triterpenic, oleanolic, and ursolic acids accounted for 93.68% ± 2.32% of the total amount of triterpene compounds in cranberry fruit samples. The composition profile of triterpene compounds in *V. oxycoccos* fruits grown under Lithuanian climatic conditions was similar to that in *V. macrocarpon* fruits [28]. A comparative analysis of the total amount of triterpene compounds showed that in *V. oxycoccos* fruit samples, it was 4060 ± 122–6542 ± 157 µg/g, whereas in *V. macrocarpon* fruit samples, it ranged from 1560.43 ± 90.81 µg/g to 4827.19 ± 91.71 µg/g [28].

Changes in the composition of bioactive compounds are determined by genetic factors [33], environmental conditions (temperature, light, humidity, and soil composition) [31], and the stage of maturity of the berries [40]. It has been found that as the berries ripen, the most significant changes occur in anthocyanins and anthocyanidins [41]. In our study, cranberry fruit samples collected in October showed higher anthocyanin content than fruit samples collected at the end of August. The coefficient of variation for anthocyanins was 62.38%. The anthocyanin content of cranberry fruit increased, on average, by 2-fold between late August and mid-October, and thus the evaluation of the stage of ripeness that determines the anthocyanin content of cranberry material is important for determining the timing of cranberry harvesting, which is of great importance in state-protected natural reserves.

The analysis of the phytochemical composition of small cranberries showed that the qualitative and quantitative composition of anthocyanins, chlorogenic acid, flavonols, proanthocyanins, and triterpene compounds found in cranberry fruit samples depended on the type of the habitat and the time of fruit collection. The phytochemical profile found in the fruit samples of small cranberry and large cranberry was virtually the same, but due to the quantitative differences in the bioactive compounds, it is expedient to standardize the phytochemical composition of cranberry raw material and the preparations produced in order to ensure the quality and effectiveness of the products. The phytochemical composition of cranberry fruit is influenced by genetic and environmental factors and the interactions between these factors, and the application of appropriate methodologies and routine tests is thus important to justify the use of cranberry fruit raw materials in the production of functional foods and phytopreparations, ensuring the control of the standardized quantitative indicators. In view of the phytochemical composition of small cranberry fruits identified in the study, the following compounds could be used as phytochemical markers: chlorogenic acid, the main anthocyanins (cyanidin-3-galactoside, cyanidin-3-arabinoside, peonidin-3-galactoside, peonidin-3-arabinoside, cyanidin-3-glucoside, and peonidin-3-glucoside), flavonols myricetin-3-galactoside and quercetin-3-galactoside, and triterpenoid ursolic and oleanolic acids.

## 4. Materials and Methods

### 4.1. Fruit Samples

**Figure 6:**
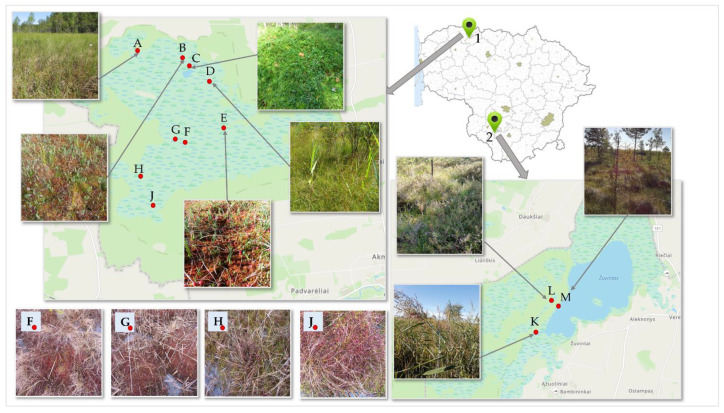
Map of cranberry fruit harvesting sites. 1—Kamanai reserve; 2—Žuvintas reserve. A description of the cranberry fruit harvesting sites is given in Table 1.

Fruit samples of small cranberries (*Vaccinium Oxycoccos* L.) were collected (300 g) in the habitats of Kamanai and Žuvintas reserves (Figure 6). The territory of Kamanai is marked in Figure 6 with number 1, and the territory of Žuvintas is marked with number 2. Cranberry samples were collected from 12 different habitats twice: in late August and in mid-October (Table 1).

Fruits were frozen at −60 °C in an ultra-low temperature freezer (CVF330/86, ClimasLab SL, Barcelona, Spain). The cranberry fruits were subsequently freeze-dried in a Zirbus lyophilizer (Zirbus Technology GmbH, Bad Grund, Germany) at a condenser temperature of −85 °C and a pressure of 0.01 mbar. Freeze-dried cranberry fruits were ground using a Retsch GM 200 electric mill (Retsch GmbH, Dreieich, Germany). Losses on drying were evaluated using the method proposed in the European Pharmacopoeia [42].

**Table 1:** Description of cranberry fruit harvesting sites.

Site	Coordinates	Altitude (m)	Collection Time	Characteristics of the Wetland Environment
A	56°18′55.3″ N 22°36′43.0″ E	81.7	1. 31 August 2021 2. 14 October 2021	Mesotrophic habitat; ass. *Caricetum lasiocarpae* Osvald 1923 em. Dierssen 1982; dominant plants: *Carex lasiocarpa, Oxycoccos palustris, Sphagnum fallax*.
B	56°18′44.2″ N 22°38′08.6″ E	81.0	1. 27 August 2021 2. 19 October 2021	Oligotrophic habitat; ass. *Sphagnetum magellanici* (Malcuit 1929) Kästner et Flössner 1933, this community is not quite typical, as it has elements of the intermediate type of swamps such as *Carex rostrata*; dominant plants: *Andromeda polifolia, Oxycoccos palustris, Carex rostrata, Sphagnum magellanicum s. l.*
C	56°18′35.7″ N 22°38′24.4″ E	83.3	1. 27 August 2021 2. 14 October 2021	Eutrophic habitat: a lot of cranberry thickets, they are concentrated on tufts, pronounced microrelief; dominant plants: *Oxycoccos palustris, Calla palustris, Eriophorum vaginatum, Lysimachia thyrsiflora, Sphagnum palustre, S. flexuosum*.
D	56°18′22.1″ N 22°39′07.4″ E	84.9	1. 31 August 2021 2. 14 October 2021	Mesotrophic habitat; ass. *Caricetum lasiocarpae* Osvald 1923 em. Dierssen 1982; dominant plants: *Carex lasiocarpa, Oxycoccos palustris, Phragmites australis, Sphagnum fallax.*
E	56°17′34.3″ N 22°39′25.4″ E	81.5	1. 30 August 2021 2. 18 October 2021	Oligotrophic habitat; ass. *Sphagnetum magellanici* (Malcuit 1929) Kästner et Flössner 1933, this community is not quite typical, as it has elements of the intermediate type of swamps such as *Carex rostrata*; dominant plants: *Andromeda polifolia, Carex rostrata, Oxycoccos palustris, Sphagnum magellanicum s. l.*
F	56°17′20.7″ N 22°37′54.2″ E	82.0	1. 30 August 2021 2. 14 October 2021	Oligotrophic habitat; ass. *Sphagnetum magellanici* (Malcuit 1929) Kästner et Flössner 1933; dominant plants: *Andromeda polifolia, Eriophorum vaginatum, Oxycoccos palustris, Phragmites australis, Sphagnum magellanicum s. l.*
G	56°17′19.7″ N 22°38′00.0″ E	82.0	1. 30 August 2021 2. 14 October 2021	Oligotrophic habitat; ass. *Sphagnetum magellanici* (Malcuit 1929) Kästner et Flössner 1933), this community is not quite typical, as it has elements of the intermediate type of swamps such as *Carex rostrata; dominant plants: Andromeda polifolia, Oxycoccos palustris, Rhynchospora alba, Sphagnum magellanicum s. l.*
H	56°16′44.4″ N 22°36′56.0″ E	79.0	1. 30 August 2021 2. 14 October 2021	Mesotrophic habitat; ass. *Caricetum lasiocarpae* Osvald 1923 em. Dierssen 1982; dominant plants: *Carex lasiocarpa, Oxycoccos palustris, Phragmites australis, Sphagnum fallax.*
J	56°16′18.4″ N 22°37′17.7″ E	81.0	1. 30 August 2021 2. 14 October 2021	Oligotrophic habitat; ass. *Sphagnetum magellanici* (Malcuit 1929) Kästner et Flössner 1933; dominant plants: *Andromeda polifolia, Oxycoccos palustris, Rhynchospora alba, Sphagnum magellanicum s. l.*
K	54°27′08.1″ N 23°35′26.1″ E	84.9	1. 31 August 2021 2. 11 October 2021	Mesotrophic habitat; *Sphagnum fallax—Eriophorum angustifolium* communities, Ass. *Caricetum lasiocarpae* Osvald 1923 em. Dierssen 1982; ass. *Scorpidio-Caricetum diandrae* Osvald 1923; ass. *Sphagno-Caricetum rostratae* Osvald 1923 em. Steffen 1931; dominant plants: *Oxycoccos palustris, Phragmites australis, Sphagnum fallax* [43].
L	54°27′49.7″ N 23°35′59.8″ E	86.0	1. 31 August 2021 2. 11 October 2021	Oligotrophic habitat; ass. *Sphagnetum magellanici* Sukopp 1959 ex Neuhäusl 1969; ass. *Sphagno tenelli-Rhynchosporetum albae* Osvald (1923) em. Dierssen 1982; dominant plants: *Oxycoccos palustris, Sphagnum fallax, Andromeda polifolia, Calluna vulgaris, Ledum palustre* [43].
M	54°27′42.4″ N 23°36′11.8″ E	86.0	1. 31 August 2021 2. 11 October 2021	Mesotrophic habitat; *Sphagnum fallax—Eriophorum angustifolium* communities, ass. *Caricetum lasiocarpae* Osvald 1923 em. Dierssen 1982; ass. *Scorpidio-Caricetum diandrae* Osvald 1923; ass. *Sphagno-Caricetum rostratae* Osvald 1923 em. Steffen 1931; dominant plants: *Oxycoccos palustris, Sphagnum fallax, Eriophorum vaginatum* [43].

### 4.2. Chemicals

All reagents, standards, and solvents were of analytical reagent grade. Ethanol 96% (*v*/*v*) was bought from AB Stumbras (Kaunas, Lithuania). Solvents (acetone, methanol, and acetonitrile), reagents (hydrochloric acid and 4-(dimethylamino) cinnamaldehyde), and reference standards (myricetin, quercetin-3-rhamnoside, quercetin-3-α-L-arabinofuranoside, quercetin-3-α-L-arabinopyranoside, oleanolic acid, β-amyrin, corosolic acid, maslinic acid, α-amyrin, and ursolic acid) were bought from Sigma-Aldrich (Steinheim, Germany). Trolox (6-hydroxy-2,5,7,8-tetramethylchroman-2-carboxylic acid) was purchased from Scharlau (Sentmenat, Spain). Formic acid was purchased from Merck (Darmstadt, Germany). The standard for quercetin-3-glucoside was purchased from Biochemistry (Buchs, Switzerland). Standards for quercetin-3-galactoside and quercetin were bought from Carl Roth (Karlsruhe, Germany). Reference standards for peonidin chloride, cyanidin chloride, myricetin-3-galactoside, malvidin-3-galactoside, malvidin chloride, malvidin-3-arabinoside, cyaniding-3-galactoside, peonidin-3-arabinoside, peonidin-3-glucoside, delphinidin-3-galactoside, cyaniding-3-glucoside peonidin-3-galactoside, and cyaniding-3-arabinoside were bought from Extrasynthese (Genay, France).

### 4.3. Sample Extraction

Extraction of proanthocyanidins, flavonols, and anthocyanins from cranberry fruits was carried out by extracting 1 g of freeze-dried cranberry powder (exact weight) with 70% ethanol acidified with 1% hydrochloric acid (*v*/*v*) in an ultrasonic bath (Elmasonic P, Elma Schmidbauer GmbH, Singen, Germany) (room temperature, 15 min at 80 kHz and 565 W). Following that, the prepared cranberry extracts were filtered into a 20 mL volumetric flask. The extraction of freeze-dried cranberry fruit samples was performed three times. The prepared cranberry extracts were kept in dark glass containers at −20 °C.

Extraction of triterpenoids from cranberries was carried out by extracting 1 g of freeze-dried cranberry fruit powder (exact weight) with 10 mL of 100% (*v*/*v*) acetone in an ultrasonic bath (Elmasonic P, Elma Schmidbauer GmbH, Singen, Germany) (temperature from 22 ± 1 °C to 60 ± 1 °C, time 60 min at 80 kHz and 1130 W). Following that, the prepared cranberry extracts were filtered into a 10 mL volumetric flask. The extraction of freeze-dried cranberry fruit samples was performed three times. The prepared cranberry extracts were kept in dark glass containers at −20 °C.

### 4.4. Spectrophotometric Technique

Freeze-dried cranberry fruit extracts were analyzed with a spectrophotometer (M550 UV/Vis, Spectronic CamSpec, Garforth, UK). We mixed 20 μL of cranberry fruit extract with 3 mL of DMCA (4-(dimethylamino)cinnamaldehyde) (0.1% DMCA (*m*/*v*) in ethanol-hydrochloric acid at 9:1 (*v*/*v*)) and measured the absorbance of this solution at 640 nm after 5 min [44]. The blank was a solution of DMCA (0.1% DMCA (*m*/*v*) in ethanol-hydrochloric acid at 9:1 (*v*/*v*)). The data were expressed in terms of (–)-epicatechin equivalent (µg/g (–)-epicatechin equivalent (EE) dry weight). The calibration equation was y = 0.7021x + 0.0138; R2 = 0.9994.

### 4.5. UPLC Separation and Quantification

Chromatographic analysis was performed using previously described developed and validated methodologies [14,45,46]. The extract analysis of cranberry fruit samples was performed using an ultra-high-performance liquid chromatography system (Waters ACQUITY UPLC, Milford, MA, USA) with a photodiode array detector (ACQUITY UPLC PDA eλ, Milford, MA, USA) and an ACE C18 reverse-phase column (100 × 2.1 mm, 1.7 µm particles) (an Avantor ACE, ACT, Aberdeen, UK). Freeze-dried cranberry fruit extracts were filtered through a filter with the pore size of 0.22 µm (Carl Roth GmbH, Karlsruhe, Germany) before injection (1 μL). The data analysis was performed using Empower (Waters) Software. The amounts of the identified compounds were calculated from the calibration curves and were expressed as µg/g dry weight.

#### 4.5.1. Analysis of Anthocyanins and Anthocyanidins

The content of anthocyanins and anthocyanidins in cranberry fruit extracts was evaluated according to the methodology described by Vilkickyte et al. [45]. The mobile phases were 100% acetonitrile (solvent A) and (*v*/*v*) aqueous 10% formic acid solution (*v*/*v*) (solvent B), and the solvent flow rate was 0.5 mL/min. The column temperature was set at 30 °C. The gradient used was 0.0–2.0 min, 95% B; 2.0–7.0 min, 91% B; 7.0–9.0 min, 88% B; 9.0–10.0 min, 75% B; 10.0–10.5 min, 20% B; 10.5–11.0 min, 20% B; and 11.0–12.0 min, 95% B. The amounts of anthocyanins and anthocyanidins were evaluated at the wavelength of 520 nm. Chromatograms of cranberry fruit samples are provided in the Supplementary Materials (Figure S1). The content of anthocyanins and anthocyanidins was calculated according to Formula (1) (where a is the amount of compound (µg/g), b is the concentration calculated from the calibration graph (µg/mL), c is the volume of the extract (mL), and d is the dry weight of the raw material (g)).

a = b × c/d (1)

#### 4.5.2. Analysis of Flavonols

The content of flavonols in cranberry fruit extracts was established according to the methodology described by Urbstaite et al. [46]. The mobile phases were 100% acetonitrile (*v*/*v*) (solvent A) and aqueous 0.1% formic acid solution (*v*/*v*) (solvent B), and the solvent flow rate was 0.5 mL/min. The column temperature was set at 30 °C. The gradient used was 0 min, 95% B; 1 min, 88% B; 3 min, 88% B; 4 min, 87% B; 9 min, 75% B; 10.5 min, 70% B; 12 min, 70% B; 12.5 min, 10% B; 13 min, 10% B; 13.5 min, 95% B; and 14.5 min, 95% B. The column was allowed to equilibrate for 2 min between the injections. The amounts of flavonols were evaluated at the wavelength of 360 nm. Chromatograms of cranberry fruit samples are provided in the Supplementary Materials (Figure S2). The amount of flavonols was calculated according to Formula (1) (where a is the amount of compound (µg/g), b is the concentration calculated from the calibration graph (µg/mL), c is the volume of the extract (mL), and d is the dry weight of the raw material (g)).

#### 4.5.3. Analysis of Triterpene Compounds

The content of triterpenoids in cranberry fruit extracts was established according to the methodology described by Sedbare et al. [14]. The mobile phases were 100% methanol (*v*/*v*) (solvent A) and aqueous 0.1% formic acid (*v*/*v*) (solvent B), and the solvent flow rate was 0.2 mL/min. The column temperature was set at 25 °C. The gradient used was 0 min, 8% B; 8 min, 3% B; 9 min, 2% B; 29.5 min, 2% B; and 30 min, 8% B. The column was allowed to equilibrate for 10 min between the injections. The amounts of triterpene compounds were evaluated at the wavelength of 205 nm. Chromatograms of cranberry fruit samples are provided in the Supplementary Materials (Figure S3). The content of triterpenoids was calculated according to the Formula (1) (where a is the amount of compound (µg/g), b is the concentration calculated from the calibration graph (µg/mL), c is the volume of the extract (ml), and d is the dry weight of the raw material (g)).

### 4.6. Statistical Design and Methods

Statistical analysis was carried out using computer software programs SPSS Statistics 21 (IBM, Armonk, NY, USA) and Microsoft Excel 2016 (Microsoft, Redmond, DC, USA). The Kruskal–Wallis one-way ANOVA test was used to evaluate differences in the content of the identified compounds between cranberry samples. Differences at *p* < 0.05 were regarded as statistically significant. The data are reported as mean ± standard deviation (SD).

## 5. Conclusions

An analysis of the phytochemical composition of small cranberry fruits grown in Lithuanian climatic conditions was conducted. Studies on the qualitative and quantitative composition of cranberry fruit samples provide new insights into the variation in the composition of anthocyanins, proanthocyanidins, chlorogenic acid, triterpene compounds, and flavonols in small cranberry fruit and enable the assessment of the quality of cranberry fruit and the use of high-quality cranberry fruit raw material for food and health promotion purposes.

In view of the phytochemical composition of small cranberry fruits found in the study, the following compounds could be used as phytochemical markers: chlorogenic acid, the main anthocyanins (cyanidin-3-galactoside, cyanidin-3-arabinoside, peonidin-3-galactoside, peonidin-3-arabinoside, cyanidin-3-glucoside, and peonidin-3-glucoside), flavonols myricetin-3-galactoside and quercetin-3-galactoside, and triterpenoid acids ursolic and oleanolic acids. A comparative analysis of the chemical composition of *V. oxycoccos* and *V. macrocarpon* fruit samples showed that there was little difference in the phytochemical profile of these plant species, but due to the quantitative differences in the bioactive compounds, it is expedient to standardize the phytochemical composition of cranberry raw material and preparations to ensure their quality and effectiveness.

## Data Availability

All data generated during this study are included in this article.

## Supplementary Materials

The following supporting information can be downloaded at: https://www.mdpi.com/article/10.3390/plants12101974/s1, Figure S1: UHPLC-PDA chromatograms of cranberry extracts (λ = 520 nm). Figure S2: UHPLC-PDA chromatogram of cranberry extracts (λ = 360 nm). Figure S3: UHPLC-PDA chromatogram of cranberry extracts (λ = 205.5 nm).
