# Supplementary material for: Phytochemical Composition of Cranberry (Vaccinium oxycoccos L.) Fruits Growing in Protected Areas of Lithuania
Source: Plants (Basel). 2023 May 13;12(10):1974. doi: 10.3390/plants12101974 (PMC10223228; doi:10.3390/plants12101974)
Supplement: Supplementary file 1 [file plants-12-01974-s001.zip › plants-2384192-supplementary.pdf]

Supplementary material:

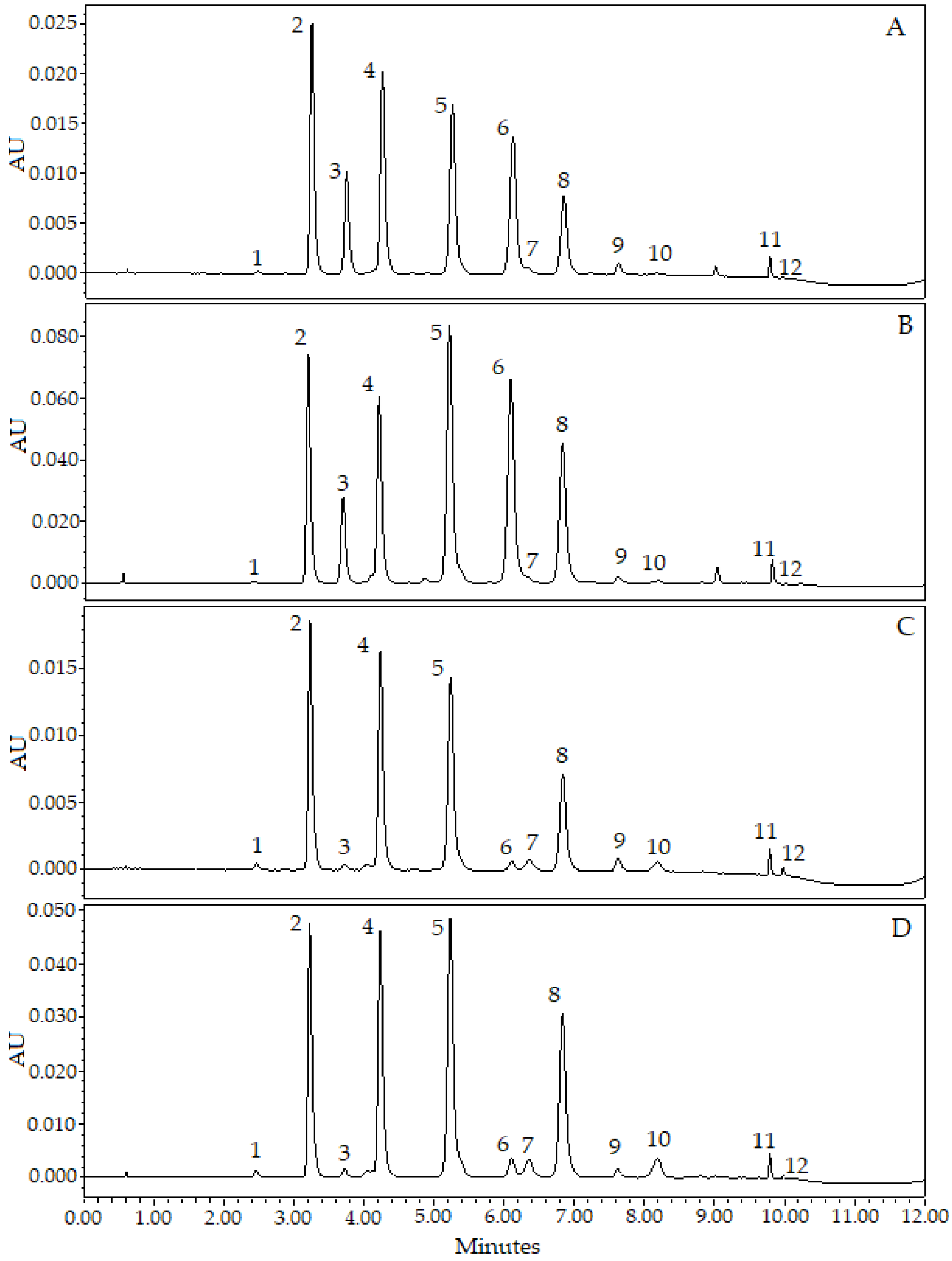

Figure S1: UHPLC-PDA chromatograms (λ = 520 nm): (A) chromatogram of *Vaccinium oxycoccas* extract prepared from fruits collected from Kamanai reserve oligotrophic (site B) wetland type at the end of August; (B) chromatogram of *Vaccinium oxycoccas* extract prepared from fruits collected from Kamanai reserve oligotrophic (site B) wetland type in October; (C) chromatogram of *Vaccinium oxycoccas* extract prepared from fruits collected from Kamanai reserve oligotrophic (site E) wetland type at the end of August; (D) chromatogram of *Vaccinium oxycoccas* extract prepared from fruits collected from Kamanai reserve oligotrophic (site E) wetland type in October. (1) Delphinidin-3-galactoside, (2) Cyanidin-3-galactoside, (3) Cyanidin-3-glucoside, (4) Cyanidin-3-arabinoside, (5) Peonidin-3-galactoside, (6) Peonidin-3-glucoside, (7) Malvidin-3-galactoside, (8) Peonidin-3-arabinoside, (9) Cyanidin, (10) Malvidin-3-arabinoside, (11) Peonidin, (12) Malvidin.

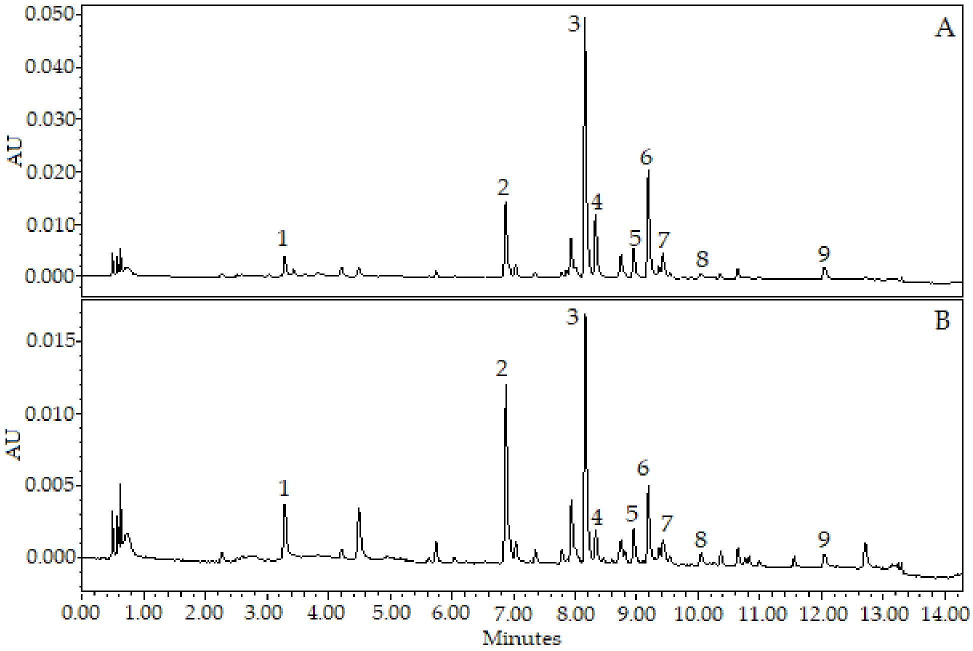

Figure S2: UHPLC-PDA chromatogram (λ = 360 nm): (A) chromatogram of *Vaccinium oxycoccas* extract prepared from fruits collected from Kamanai reserve mesotrophic (site D) wetland type at the end of August; (B) chromatogram of *Vaccinium oxycoccas* extract prepared from fruits collected from Kamanai reserve mesotrophic (site D) wetland type in October. (1) chlorogenic acid, (2) myricetin-3-galactoside, (3) quercetin-3-galactoside, (4) quercetin-3-glucoside, (5) quercetin-3-α-L-arabinopyranoside, (6) quercetin-3-α-L-arabinofuranoside, (7) quercetin-3-rhamnoside, (8) myricetin, (9) quercetin.

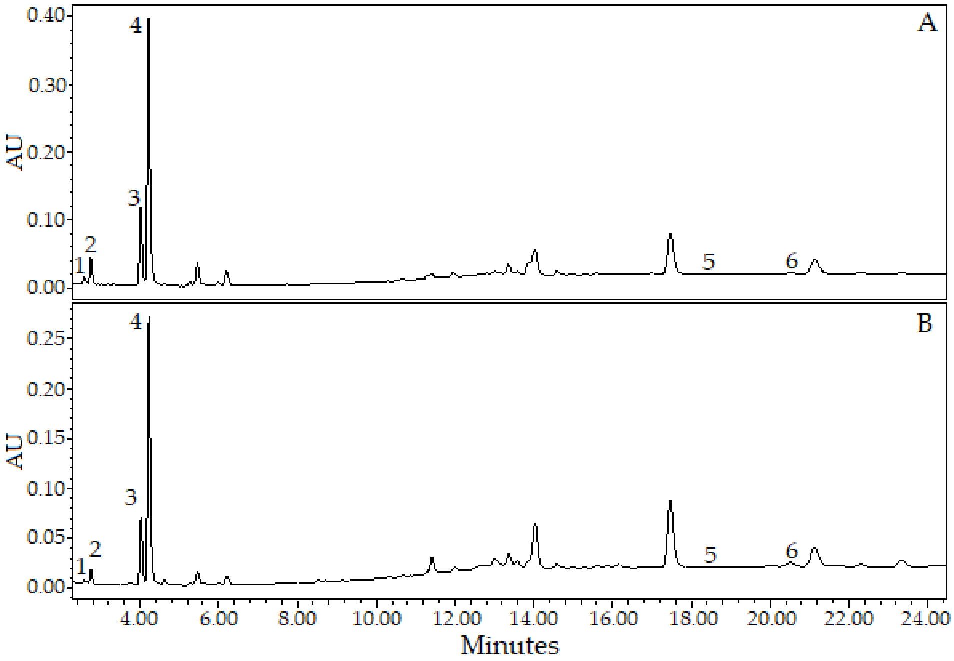

Figure S3: UHPLC-PDA chromatogram (λ = 205.5 nm): (A) chromatogram of *Vaccinium oxycoccas* extract prepared from fruits collected from Kamanai reserve mesotrophic (site D) wetland type at the end of August; (B) Chromatogram of *Vaccinium oxycoccas* extract prepared from fruits collected from Kamanai reserve mesotrophic (site D) wetland type in October. (1) maslinic acid, (2) corosolic acid, (3) oleanolic acid, (4) ursolic acid, (5) β-amyrin, (6) α-amyrin.
